# *Klebsiella pneumoniae* Liver Abscess and Metastatic Endophthalmitis

**DOI:** 10.1177/2324709615624125

**Published:** 2015-12-31

**Authors:** Jason T. Wells, Catherine R. Lewis, Omar K. Danner, Kenneth L. Wilson, L. Ray Matthews

**Affiliations:** 1 Greenville Health System, Greenville, SC, USA; 2 Morehouse School of Medicine, Atlanta, GA, USA

**Keywords:** *Klebsiella pneumoniae*, liver abscess, metastatic endophthalmitis, percutaneous drainage

## Abstract

*Introduction. Klebsiella pneumoniae* is a well-known cause of liver abscess. Higher rates of liver abscess associated with *Klebsiella pneumoniae* are seen in Taiwan. Metastatic endophthalmitis is a common complication associated with a poor prognosis despite aggressive therapy. *Case Report*. We report a case of a 67-year-old Korean female with *Klebsiella pneumoniae* liver abscess. The patient developed metastatic endophthalmitis and ultimately succumbed to her disease despite aggressive medical and surgical treatment. *Conclusion*. Dissemination of *Klebsiella pneumoniae* is associated with significant morbidity and mortality. Liver abscesses preferably should be treated with percutaneous drainage, but surgical treatment is needed in some cases. Metastatic spread to the eye is a common complication that must be treated aggressively with intravenous antibiotics and surgical intervention if necessary.

## Introduction

*Klebsiella pneumoniae* has been implicated as a common cause of infection in the human body. The liver is a common source of infection, either due to a primary hepatobiliary disease or secondary to an intra-abdominal infection. Although rare, there is significant mortality and morbidity associated with this organism.

## Case Report

**Figure 1.**
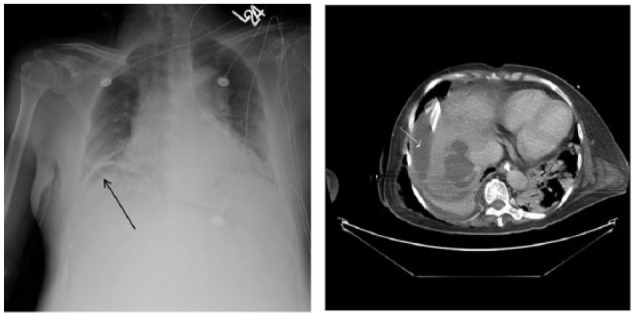
(A) Chest x-ray demonstrating a crescentic lucency underneath the right hemidiaphragm concerning for free air within the abdomen (black arrow). (B) Large right liver abscess (white arrow).

This patient was a 67-year-old previously healthy Korean female with no significant past medical or surgical history, who presented with a 7-day history of progressively worsening abdominal pain. She had recently come to America from South Korea to stay with her daughter about 13 months ago. The patient’s vital signs were within normal limits. Abdominal examination was significant for diffuse tenderness to palpation, distention, and voluntary guarding. White blood cell count was 16 000 /µL and alkaline phosphatase was 133 U/L. Computed tomography (CT) scan of the abdomen showed a large hepatic abscess noted in the right lobe of the liver (Figure 1).

**Figure 2.**
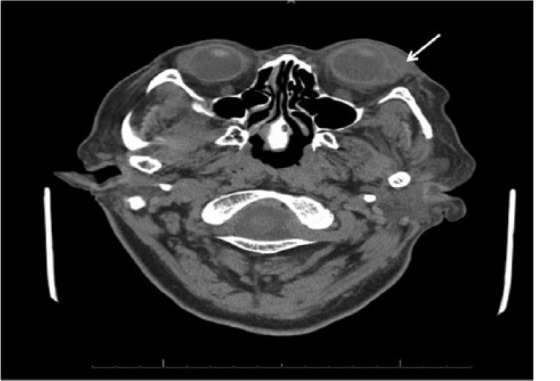
Abnormal intraocular enhancement of the left globe involving both the posterior and anterior chambers consistent with active endophthalmitis (arrow).

The patient was treated aggressively for septic shock over the next several days with vancomycin and zosyn. The patient was taken to the operating room for an exploratory laparotomy for likely perforated hepatic abscess. A large hepatic abscess was noted within the right lobe, with free suppurative fluid in the abdomen. There was an extensive amount of fibrinous exudute present within the abdominal cavity, which was irrigated thoroughly with normal saline. The abscess cavity was opened and Penrose drains were placed into the abscess cavity and brought out through the skin to allow for drainage. The abdomen was closed in the standard fashion. Postoperatively, the patient was admitted to the surgical intensive care unit and treated for septic shock. Intra-abdominal cultures taken during the surgery were positive for *Klebsiella pneumonia*e. Despite intravenous antibiotics therapy, the patient continued to have severe sepsis. Infectious Disease services were consulted to assist in the patient’s management. Her antibiotic course was later changed to imipenem, vancomycin, and fluconazole due to the development of microbial resistance. On postoperative day 7, the patient developed swelling and purulent drainage from the left eye. A CT scan of the orbits showed a detached retina on the left with opacification and endophthalmitis (Figure 2).

Intravitreal injections of vancomycin and ceftazidime antibiotics were given for 8 days and the patient was subsequently taken to the operating room for enucleation and washout by the ophthalmology service. Pathology was consistent with Panophthalmitis. Tissue cultures of the eye, optic nerve, and bronchial washings were also positive for *Klebsiella pneumonia*e. Despite aggressive medical and surgical efforts, the patient eventually succumbed to multiorgan system failure from septic shock on postoperative day 45.

## Discussion

The most common location for a visceral abscess is the liver. The incidence of liver abscesses depends on the population studied. The rate can be estimated at 2.3 cases per 100 000 people, with a higher incidence in men versus women (3.3 to 1.3 per 100 000). Higher rates are reported in Taiwan at 17.6 cases per 100 000.^1-4^ Although the mortality rate is relatively low, the prognosis in patients who develop metastatic endophthalmitis is poor despite aggressive therapy, as many patients develop impaired vision or blindness.^5^ While rare, this condition has significant morbidity that depends on the type and source of the primary infection.

Most incidences of metastatic infection are reported in Asia.^5-7^ At least one retrospective review in the United States examined 79 cases of liver abscess during which *Klebsiella pneumoniae* was the most commonly found pathogen in 43% of patients.^8^ Primary *Klebsiella* liver abscesses commonly occur in diabetics, but other comorbidities can increase the risk of this infection.^7,9-11^

The most common clinical manifestations of a primary liver abscess include fever, right upper quadrant tenderness, nausea, vomiting, diarrhea, leukocytosis, and elevated alanine aminotransferase, aspartate aminotransferase, alkaline phosphatase and bilirubin.^7^ Approximately 13% of patients with a primary liver abscess develop metastatic infections, with the most common manifestations being endophthalmitis, meningitis, and cerebral abscess.^5,7,9,12^ *Klebsiella pneumoniae* is the most common bacterial isolate to cause metastatic infections.^9^

The imaging modalities of choice for diagnosis of a liver abscess are ultrasonography and CT. Once the area of infection has been identified, the preferred method of treatment is percutaneous drainage and intravenous antibiotics. Initial antibiotic treatment should be empiric until sensitivities and specificities have been obtained. The duration of antibiotics should be a total of 4 to 6 weeks. Studies show that abscesses <5 cm in diameter can be treated with simple aspiration and/or by leaving a drainage catheter in place until the output is minimal. For lesions >5 cm, surgical intervention is preferred.^13^ In this case, emergent surgical exploration was warranted due to peritonitis and perforation.

## Conclusion

*Klebsiella pneumoniae* dissemination is associated with significant morbidity and mortality. A high index of suspicion for metastatic spread to various other organs including the eye is necessary. Because of the frequency of *Klebsiella*-associated liver abscesses in the Asian population, treatment of *Klebsiella* species should begin until proven otherwise. Early detection of *Klebsiella*-associated endophthalmitis and prompt treatment with aggressive intravenous antibiotics may be the only method to salvage visual acuity and decrease the incidence of overall morbidity and mortality.
